# PDMS-ZnO Piezoelectric Nanocomposites for Pressure Sensors

**DOI:** 10.3390/s21175873

**Published:** 2021-08-31

**Authors:** Karina Jeronimo, Vasileios Koutsos, Rebecca Cheung, Enrico Mastropaolo

**Affiliations:** School of Engineering, The University of Edinburgh, Sanderson Building, Robert Stevenson Road, The King’s Buildings, Edinburgh EH9 3FB, UK; k.jeronimo@ed.ac.uk

**Keywords:** polydimethylsiloxane, zinc oxide, thermomechanical characterisation, piezoelectric properties, nanocomposites, sensors

## Abstract

The addition of piezoelectric zinc oxide (ZnO) fillers into a flexible polymer matrix has emerged as potential piezocomposite materials that can be used for applications such as energy harvesters and pressure sensors. A simple approach for the fabrication of PDMS-ZnO piezoelectric nanocomposites based on two ZnO fillers: nanoparticles (NP) and nanoflowers (NF) is presented in this paper. The effect of the ZnO fillers’ geometry and size on the thermal, mechanical and piezoelectric properties is discussed. The sensors were fabricated in a sandwich-like structure using aluminium (Al) thin films as top and bottom electrodes. Piezocomposites at a concentration of 10% *w*/*w* showed good flexibility, generating a piezoelectric response under compression force. The NF piezocomposites showed the highest piezoelectric response compared to the NP piezocomposites due to their geometric connectivity. The piezoelectric compound NF generated 4.2 V while the NP generated 1.86 V under around 36 kPa pressure. The data also show that the generated voltage increases with increasing applied force regardless of the type of filler.

## 1. Introduction

Recent developments in the field of flexible electronics have increased the exploration of a new class of emerging materials such as piezocomposite materials [1,2,3]. The fabrication of piezocomposite materials has an increasing interest due to the combination of two materials with different properties such as the mechanical flexibility provided by the polymer and the piezoelectricity given by the fillers involved. These materials are generally fabricated using a low-cost and simple process under low temperatures with enhanced properties that can be tailored towards specific applications [4]. However, the challenges remain when it comes to the piezoelectric properties of these nanocomposites as it will depend on the type of fillers used and the connectivity between these fillers which can be strongly affected by different factors such as the fabrication method used, filler concentration, size and shape of fillers, dispersion and distribution of fillers and polymer–filler interactions. On the other hand, the integration of the fillers into the polymer matrix can also have an impact on other properties including mechanical and thermal [5,6,7,8].

Poly(dimethylsiloxane) (PDMS) has attracted immense interest within the scientific and industrial community in a wide range of fields including electronics, medical devices, adhesives, robotics and coatings. PDMS exhibits interesting properties such as flexibility, hydrophobicity, chemical stability, biocompatibility and high resistance to thermal and thermo-oxidative degradation in a wide temperature range (−100–300 °C) [4,9,10]. In order to harness these properties as well as converting it into a piezoelectric material, a variety of piezoelectric fillers have been integrated into the polymer. Piezoelectric materials such as lead zirconate titanate (PZT), barium titanate (BaTiO_3_), aluminium nitride (AlN), and gallium nitride (GaN) have been widely used in the field of micro-electromechanicalsystems (MEMS) and nano-electromechanicalsystems (NEMS) and sensors [11,12]. However, the use of these materials require expensive and complicated fabrication processes and most of them are not considered biocompatible nor eco-friendly materials. Therefore, zinc oxide (ZnO) is preferred over the above mentioned piezoelectric materials due to its simple fabrication and non-toxic properties. ZnO has the richest family of nanostructures in comparison to other materials as it can be obtained as wires, rods, flowers, belts, helixes, walls, etc [3,13,14,15,16]. Studies have reported improvement in toughness, scratch resistance, resistance to fatigue, thermal stability, weatherability and optical and barrier properties when integrating ZnO nanowires and nanoparticles into polymers enabling a wide range of applications [17,18]. The integration of ZnO fillers into PDMS has been reported to generate a piezoelectric response, being biocompatible and also environmentally friendly, making them non-toxic and safe to be disposed of in a landfill after use [3,19,20,21,22]. Among the ZnO nanomaterials, nanoparticles and nanowires have been commonly used in nanocomposites but fewer studies have used other types of nanostructures [14,23,24]. 3D nanostructures such as ZnO NFs have been found to enhance light harvesting, photocatalytic performance and antibacterial activity; this is due to their high surface area to volume ratio, surface-reaction efficiency and better charge transfer and carrier immobility [25]. Among the ZnO NFs, different type of morphologies have been reported such as ones with cabbage-like structures and petal-like structures resulting in a microstructural arrangement composed of many nanostructures [26,27,28]. The piezoelectric features of ZnO fillers combined with the flexibility of PDMS can pave the way towards potential piezocomposite materials for wearable/implantable biomedical devices and electronic skins [1,3,19,20,21,22,29,30,31].

The size and shape of the fillers together with the concentration are crucial parameters to reach a piezoelectric response when embedded in a continuous polymer matrix. The interphase region in which the filler is surrounded by the polymer can have a significant effect on the final properties of the nanocomposite. Therefore, the use of 3D nanostructures have become more attractive candidates for piezoelectric fillers due to their geometry, size and spatial distribution. To the best of our knowledge, the majority of the studies have been dedicated mainly to ZnO particles and nanowires embedded in polymers but only a few have used different fillers to compare the effect of the geometry and size in their piezoelectric response [17,28,32]. For instance, the addition of ZnO micron-sized fillers in PDMS as piezocomposite material for energy harvesting and biosensors using high concentrations of 30% by weight have been reported to increase the Young’s modulus (less flexibility) [33]. Another study reported that there is a direct correlation between filler concentration and output voltage obtaining the highest output voltage of 23 V when subjected to a load resistance of 10 MΩ at 7.4% wt concentration of ZnO nanoparticles into PDMS ranging from 2% wt to 9.1% wt concentrations [8]. Recently, a composite film of PDMS and ZnO tetrapods was reported to be potentially used as piezocapacitive pressure sensor with a sensitivity of 2.55 kPa^−1^ [24].

Therefore, it is important to characterise whether the addition of the ZnO nanofillers into a PDMS matrix compromises the flexibility, thermal stability and conformability when reaching a piezoelectric percolated network of fillers. Despite the amount of effort looking for other piezocomposites alternatives, researchers have reported difficulties in regards to the processability, limitation of operating temperatures and the need for additional steps such as poling, dielectrophoresis or doping techniques. This work is dedicated to the design, fabrication and characterisation of PDMS-ZnO nanocomposites using in-house synthesised dendritic nanoflowers and commercially available nanoparticles. The effect of size and geometry connectivity of the fillers on the mechanical and thermomechanical properties has been analysed by performing uniaxial tensile testing, dynamic mechanical analysis and thermogravimetric analysis. Finally, in order to test the potential of the piezoelectric properties of the nanocomposites up to 10% wt concentration of ZnO fillers, the specimen was subjected to different loads while the electrical response was recorded.

## 2. Materials and Methods

### 2.1. Fabrication of PDMS-ZnO Nanocomposites

#### 2.1.1. ZnO Nanofillers

Two different ZnO nanofillers were used: in-house synthesised ZnO NFs and commercially NPs purchased from Sigma–Aldrich (size < 100 nm). The in-house ZnO NFs were synthesised via a hydrothermal method which consisted of the reaction of zinc nitrate Zn(NO_3_)_2_·6H_2_O and hexamethylenetetramine (HMTA) in de-ionized water mixed at an equimolar concentration (20 mM). The zinc nitrate and HMTA were added in de-ionized water and magnetically stirred for 10 min. The solution was sealed and kept in the oven at 90 °C for 18 h. Once the solution was cooled, it was further washed and centrifuged 3 times with ethanol. Finally, the remaining sediment (white powder) in ethanol was left to dry at room temperature overnight.

#### 2.1.2. PDMS-ZnO Nanocomposites

The nanocomposites were fabricated by mixing the PDMS base polymer and the ZnO nanofillers at different concentrations (0.1, 0.3, 0.5, 0.7, 1, 2, 3 and 5 % *w*/*w).* Each mixture was magnetically stirred for 10 min to minimise the formation of large agglomerates within the material. Then, the curing agent was added in a ratio of 10:1 followed by additional magnetic stirring for 15 min to obtain a better dispersion within the mixture. Finally, the mixture was poured into an aluminium mould and degassed for 2 h in a vacuum desiccator in order to remove the air bubbles resulting from the stirring process. The samples were cured at 100 °C for 60 min in a pre-heated oven. Two PDMS-ZnO nanocomposites batches were fabricated including the in-house synthesised NFs (PDMS-NFs) and the purchased NPs (PDMS-NPs). Each batch consisted of 40 samples (5 identical samples for each concentration).

#### 2.1.3. Fabrication of PDMS Nanocomposites-Based Sensors

**Figure 1:**
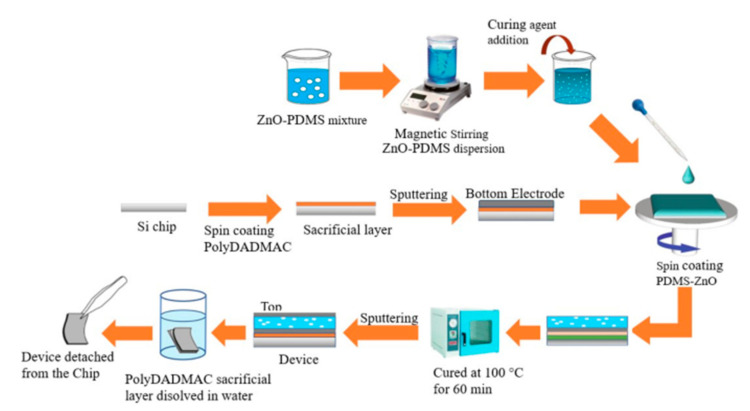
The fabrication process of PDMS piezocomposite sensors.

Square chips with dimensions of 40 mm × 40 mm were used as carrier chips. Poly(diallyl dimethylammonium chloride) (PolyDADMAC) was spin-coated onto the carrier chip as a sacrificial layer followed by a deposition of a 500 nm layer of aluminium (Al) which acts as the bottom electrode. PDMS-ZnO mixtures of 5 and 10% *w*/*w* were spin-coated on top of the Al layer. Then, the chips were cured at 100 °C for 60 min followed by another deposition of a 500 nm thick Al layer to act as the top electrode. Finally, the chips were immersed in water for around 4 h in order to completely dissolve the PolyDADMAC sacrificial layer and the PDMS-ZnO nanocomposite devices were then fully released from the carrier chip. Finally, Al foil strips were connected to the top and bottom electrodes using Ag adhesive paste. A schematic diagram of the fabrication process is shown in Figure 1.

### 2.2. Characterisation and Testing Methods

#### 2.2.1. Tensile Testing

**Figure 2:**
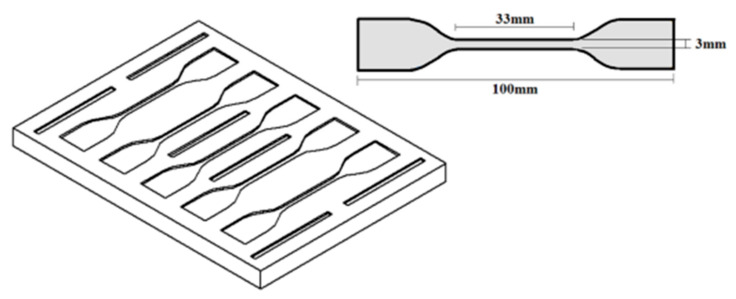
Aluminium mould—ASTM D412/dog-bone shaped samples.

The mechanical properties of the nanocomposites were determined by tensile testing performed with a universal tensile machine (INSTRON 3367, Norwood, MA, USA) fitted with a load cell of 50 N at a rate of 50 mm/min. Each set contained 5 dog-bone shaped samples with a gauge length of 33 mm, a thickness of 1 mm, and a cross-sectional area of 3 mm × 1 mm (ASTM D412 standards) (Figure 2).

#### 2.2.2. Scanning Electron Microscopy (SEM)

Microstructural characterisation of the ZnO nanofillers used (NFs and NPs), PDMS-NPs and PDMS-NFs nanocomposites was carried out by a thermionic emission electron microscope (TESCAN-VEGA3). Fractured surfaces of the nanocomposites samples after tensile testing were investigated to obtain information about the dispersion of nanofillers within the silicone matrix and the adhesion between polymer and nanofillers. In this case, the fractured surfaces were sputter-coated with a thin film of gold to facilitate scanning electron microscopy (SEM) imaging.

#### 2.2.3. X-ray Diffraction (XRD)

The crystal structures of the ZnO nanostructures (NFs and NPs) were characterised by X-ray diffraction (Bruker D8 Advance) using Cu Kα radiation (λ = 1.54018 Å) in the range between 30° and 70° operated at a voltage of 40 kV and a current of 40 mA.

#### 2.2.4. Fourier Transform Infrared Spectroscopy (FTIR)

A Perkin Elmer Spectrum65 FT-IR Spectrometer was employed to investigate the interaction between the PDMS and the ZnO nanofillers. The spectra of neat PDMS and PDMS-ZnO nanocomposites (5% *w*/*w*) were obtained in the range between 500 cm^−1^ to 4000 cm^−1^.

#### 2.2.5. Brunauer, Emmett and Teller (BET) Surface Areas

The surface characterisation area of the ZnO nanostructures was estimated using a Quantachrome Quadrasorbevo (Boynton Beach, FL, USA) automated surface area analyser. The measurements were carried out by the adsorption/desorption of low-temperature (77 K) nitrogen and carbon dioxide (273 K) as a function of relative pressure. The weight of the powdered samples was approximately 100 mg and they were degassed for 16 h at 125 °C under a high vacuum prior to analysis.

#### 2.2.6. Dynamic Mechanical Analysis (DMA)

The dynamic mechanical data of the nanocomposites measurements were obtained by using a DMA (Q8000) of TA instruments. Samples (10 mm × 10 mm × 2 mm) were tested on a tensile mode at a constant frequency of 1 Hz in a temperature range from −150 °C to 150 °C at a scanning rate of 3 °C/min. Storage modulus (E’) and loss tangent (tanδ) were determined as a function of temperature for neat PDMS and PDMS-ZnO nanocomposites (NFs and NPs). The maximum peak of the loss tangent as a function of temperature corresponds to the glass–rubber transition temperature (Tg) of the material (neat PDMS or PDMS-ZnO nanocomposite).

#### 2.2.7. Thermogravimetric Analysis (TGA)

The thermal stability of the nanocomposites was analysed by performing the thermogravimetric analysis (TGA) using an SDT Q600 from TA Instruments. The neat PDMS and PDMS-ZnO nanocomposites (NFs and NPs) samples were heated at a rate of 10 °C/min from 30 to 800 °C in an N_2_ atmosphere.

#### 2.2.8. Piezoelectric Testing of Nanocomposites

**Figure 3:**
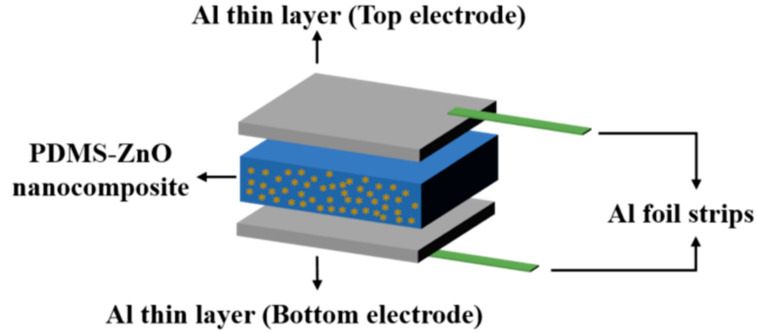
Schematic of fabricated PDMS piezocomposite sensor.

The piezoelectric response of the PDMS piezocomposites sensors has been characterised by applying a dynamic load using a motorised test stand (Mark-10 ESM303, Coplague, NY, USA) which is comprised of a flat and circular crosshead with a diameter of 10 mm. The top and bottom electrodes of the sensors have been connected to a nanovoltmeter (Keithley 2182A, Beaverton, OR, USA) and tested to different compressive pressures. The voltage between the top and bottom electrodes has been reported when applying pressure for 0.5 s and then released, repeating the process five times with a 0.5 s pause between each cycle. A schematic diagram of the fabricated PDMS piezocomposites sensors is shown in Figure 3.

## 3. Results and Discussion

### 3.1. Microstructural Characterisation

**Figure 4:**
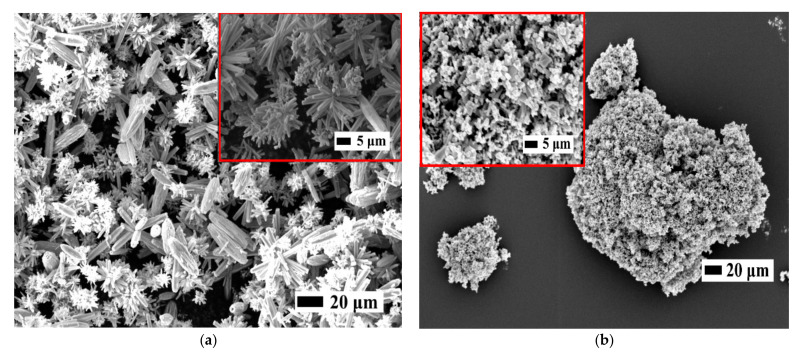
SEM images of (**a**) in-house synthesised dendritic ZnO NFs and (**b**) purchased agglomerated ZnO NPs.

The structural properties of the ZnO fillers (purchased NPs and in-house synthesised NFs) used in the piezocomposites such as shape, size, morphology and crystal size were analysed by SEM, XRD and BET analysis, respectively. Figure 4a,b show the SEM images of the ZnO fillers in the form of powder of in-house synthesised dendritic NFs and purchased NPs ones, respectively.

In Figure 4a, the in-house synthesised particles show a flower-like microstructure formed by individual nanorods grown radially from a centre in which these exhibit different lengths within 3 and 6 μm while the diameters are between 180–220 nm (i.e., dendritic NFs). On the other hand, the purchased NPs are shown in Figure 4b (diameter < 100 nm, rhombohedral shape) exhibiting an irregular shape in agglomerates of different sizes (5–30 μm).

**Figure 5:**
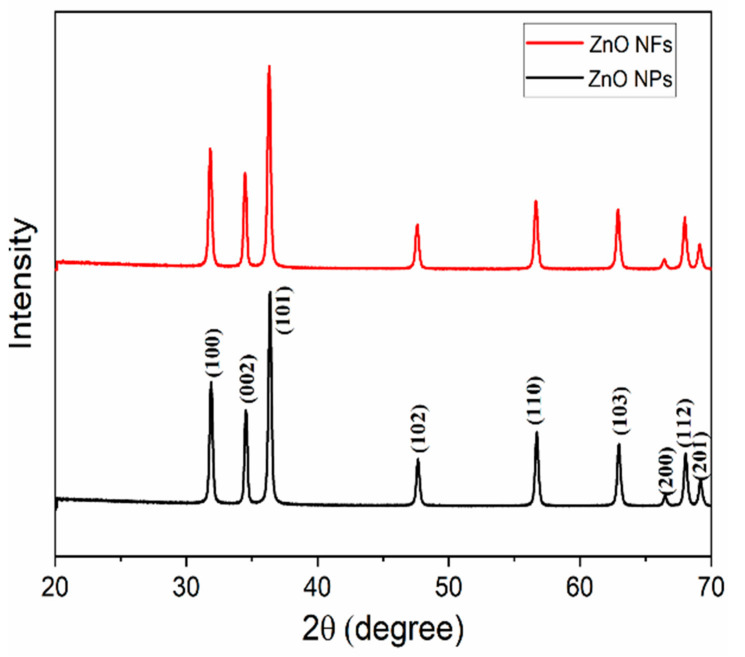
XRD patterns of the ZnO nanostructures: NPs and NFs.

The structural properties of the ZnO nanostructures used were investigated by XRD and BET. Figure 5 shows the diffraction patterns of the NPs and NFs, respectively. The diffraction peaks at 2θ: 31.9, 34.53, 36.38, 47.65, 56.71, 62.98, 66.50, 68.03 and 68.19 correspond to (100), (002), (101), (102), (110), (103), (200), (112) and (201) crystal planes are associated to the hexagonal wurtzite crystal structure of ZnO and were in agreement with the Joint Committee on Powder Diffraction Standards (JCPDS)—International Centre for Diffraction Data card No. 36-1451 [34]. Therefore, one can confirm that the in-house synthesised ZnO NFs are free of impurities and exhibit good crystallinity compared to the commercial and standard database.

The average crystal sizes of the particles were determined using the Debye–Scherrer Equation (1):

(1) $$D=\frac{K\lambda }{FWHM\;cos\theta }$$

where *D* is the crystal size, *K* is the Scherrer constant which is related to the crystallite shape and is usually 0.9, *λ* is the wavelength of light used for diffraction (CuKα) which is 1.54 Å, *FWHM* (full width at half maximum) of the diffraction peak and *θ* is the angle of diffraction. The average size of the particle was found to be 32.95 nm and 32.65 nm for NPs and NFs, respectively.

In addition, the surface area of the ZnO NPs and NFs was obtained from the BET analysis shown in Table 1. The surface area of NPs is almost 6 times higher than the NFs which means that NPs are smaller than the NFs as seen in the SEM images. Therefore, dispersion and distribution can be more difficult to achieve when using NPs rather than NFs. The average crystalline size of the particles was also calculated by using the following Equation (2):

(2) $$d_{BET}=\frac{6000}{\rho S_{BET}}$$

where *d_BET_* corresponds to the crystalline size (nm), *ρ* is the density of ZnO powder (g/cm^3^) and *S_BET_* is the BET surface area (m^2^/g). The values of the crystalline sizes are shown in Table 1 showing that NPs is 76.50 nm while NFs is 486.58 nm. The difference between the values obtained by BET and XRD is probably an indication of a conglomeration of the ZnO powders which is in agreement with other studies [34]. Therefore, the agglomeration coefficient was determined by using the following Equation (3):

(3) $$C_{F}=\frac{d_{BET}}{D}$$

where *C_F_* corresponds to the agglomeration coefficient, *d_BET_* is the crystalline size (nm) and *D* is the average crystalline size of the ZnO. The *C_F_* obtained for NPs is 1.39 while for NFs is 1.87 indicating that the agglomeration is bigger for NFs than for NPs prior to the mixing process for the fabrication of the nanocomposite.

### 3.2. Mechanical Characterisation

**Figure 6:**
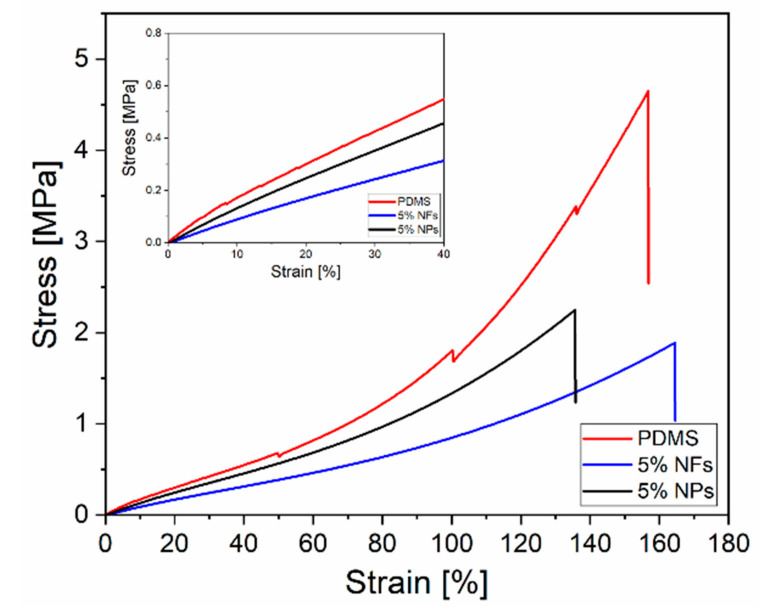
Stress–strain curves obtained from tensile tests for PDMS, PDMS-5%ZnO NPs and PDMS-5%ZnO NFs. Inset: stress–strain curves up to 40% strain.

The Young’s modulus (*E*) has been calculated using the stress-strain curves up to 40% strain. When analysing mechanical properties of rubbers, *E* is calculated in the 0–40% strain interval (small deformations for rubbers) thus providing information about the stiffness of the material in this interval [4,35,36]. Other parameters, including elongation at break (Eb%) and ultimate tensile strength (*UTS*) (i.e., the maximum stress that a material can withstand load just before failure), were measured to explore the way nanofillers affect the mechanical properties of the initial polymer. Each value corresponds to the mean of five measurements with its respective standard deviation. Volume fraction, aspect ratio, polymer–filler interactions, orientation and distribution of the nanofillers must be considered to shed light on the changes seen in the bulk mechanical properties of the initial material. Figure 6 shows some of the original stress–strain curves until sample failure obtained from the tensile testing for PDMS, PDMS-5%NFs and PDMS-5%NPs, respectively.

**Figure 7:**
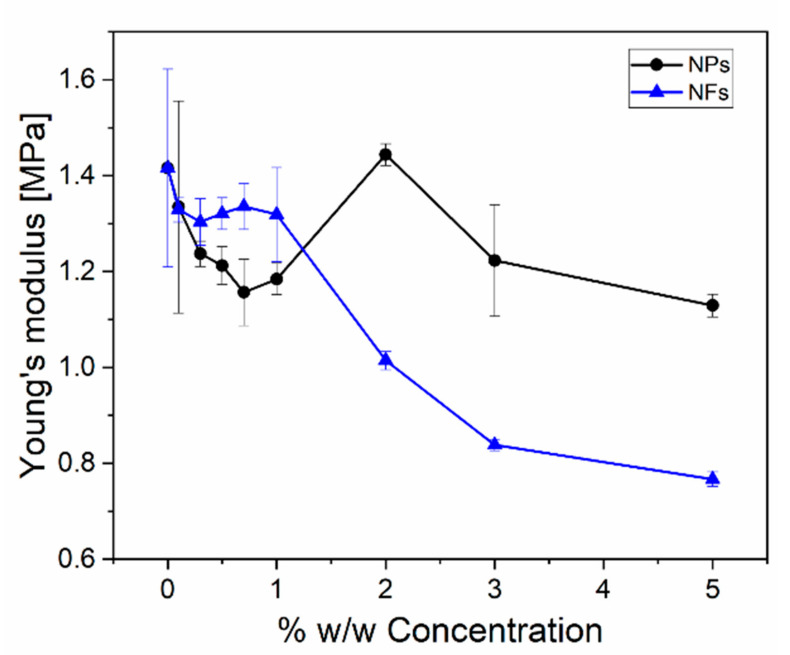
Young’s modulus of PDMS-NPs and PDMS-NFs as a function of nanofillers concentrations.

Figure 7 shows *E* values as a function of NFs and NPs loading concentrations obtained from the tensile testing of the piezocomposites up to 40% strain. At low loadings (<1%) the elastic modulus *E* showed nearly the same value as for neat PDMS (1.42 ± 0.21MPa) regardless of the type of filler, while at higher loadings (5%) *E* decreased to 0.77 ± 0.015 for NFs and 1.13 ± 0.02 for NPs. At this concentration, the formation of agglomerations is low and the mechanical properties are not significantly affected while for concentrations from 2% up to 5%, *E* was found to decrease regardless of the type of filler. The observed decrease can be explained by the fact that nanofillers can restrict the formation of the 3D networks within the polymer, a weak filler–matrix interaction and an increase in the interacting interphase region [37,38,39,40,41]. The introduction of ZnO nanofillers in the PDMS matrix inhibits the curing process, thus resulting in a decrease in crosslinking density leading to a final nanocomposite with a lower *E* [42,43]. As concentration increases, filler–filler interactions seem to be more predominant upon matrix–filler interactions, thus, showing a lack of physical and chemical interaction between PDMS-ZnO which is correlated with the FTIR analysis in the next section [44]. Low molecular weight PDMS such as Sylgard 184 has been found to be immiscible with ZnO fillers leading to the formation of macroscopic phase separations [16]. However, the observed decrease in *E* by 20% means that the produced piezocomposites still exhibit the desirable flexibility required for flexible sensor applications [4].

It is worth pointing out that the PDMS-NPs exhibits slightly higher *E* than PDMS-NFs for loadings >1% probably due to better interfacial adhesion as mentioned before. In this respect, the difference in *E* between the two different nanocomposites (PDMS-NPs and PDMS-NFs) might be due to the difference in the fillers shape, surface area and roughness. Furthermore, *E* can be affected also by the degree of dispersion and aggregation of the nanofillers within the polymer matrix. By using only direct mechanical mixing, uniform dispersion is difficult to achieve since van der Waals forces between fillers are most likely dominating over the interaction forces between fillers and polymers. Under these conditions, fillers agglomerates are formed thus affecting *E*. The formation of the agglomerates affects the interphase region which has been shown to affect the properties of the nanocomposites [8].

**Figure 8:**
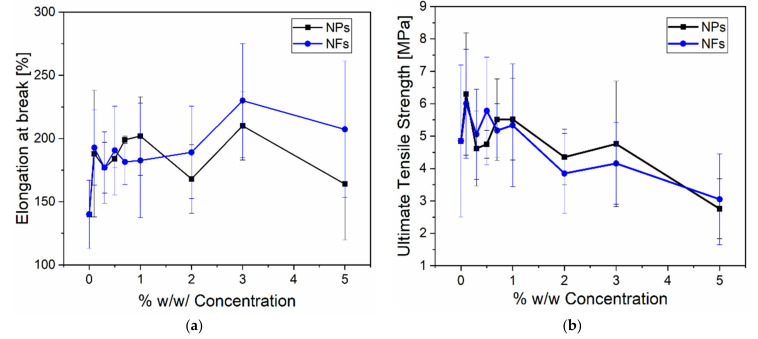
Elongation at break (Eb%) and ultimate tensile strength (UTS) as a function of concentration for (**a**) PDMS-NPs and (**b**) PDMS-NFs.

Figure 8 shows the elongation at break (Eb%) and ultimate tensile strength (UTS) as a function of nanofillers concentration. Figure 8a shows a slight increase in Eb% when introducing nanofillers. Figure 8b shows no influence on the UTS as the nanofillers concentration increases. Therefore, the nanocomposites show a softening effect when introducing the ZnO nanofillers enabling more deformation of the PDMS matrix and less stress transferred to the fillers. Similarly, this can be explained by the lack of interaction between polymer and filler and the weak correlation of Eb% and UTS with concentration. However, the slight increase in Eb% is probably due to wetting properties and strong interfacial adhesion [20].

**Figure 9:**
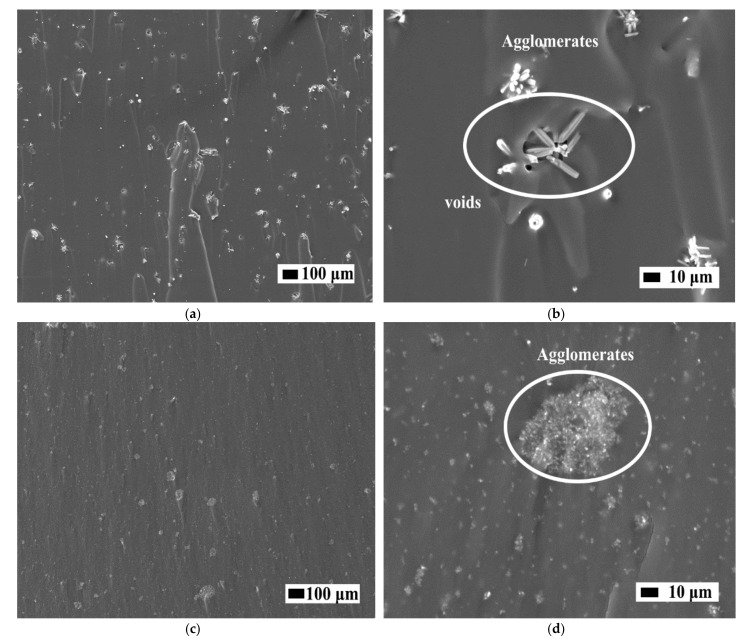
SEM images of fractured surface: (**a**,**b**) 5% PDMS-NFs, (**c**,**d**) 5% PDMS-NPs nanocomposite materials.

The fractured surface area of the nanocomposites at 5 %wt concentration of NFs and NPs are shown in Figure 9. The presence of micron-sized agglomerates on the fractured surfaces (after the tensile test) of the samples regardless of the use of NFs or NPs as nanofillers can be observed. ZnO NFs randomly distributed seem to be pulled out from the surface (holes) indicating poor adhesion between the nanofiller and the polymer matrix while NPs appear to have a stronger adhesion with the polymer matrix probably due to their smoother surface, size and area leading to better wetting properties and interfacial adhesion.

Even though the *C_F_* was shown to be bigger for NFs than NPs, it can be observed that after mixing, the aggregation of NFs is less compared to NPs due to their surface area, size and random orientation (i.e., pointing in different directions). Thus, the NFs can be better dispersed under a mechanical mixing process compared to the NPs. The distance between NPs is shortened due to their higher surface area and filler-filler interaction resulting in the formation of microscale agglomerates. It is important to mention that in this work no special coupling agent was used in our ZnO fillers. Therefore, in both nanocomposites, one can assume that the chemical interaction between polymer-filler (PDMS-ZnO) is the same while the interfacial adhesion at the interphase changes based on the surface area, shape and size of the fillers (see Table 1).

**Figure 10:**
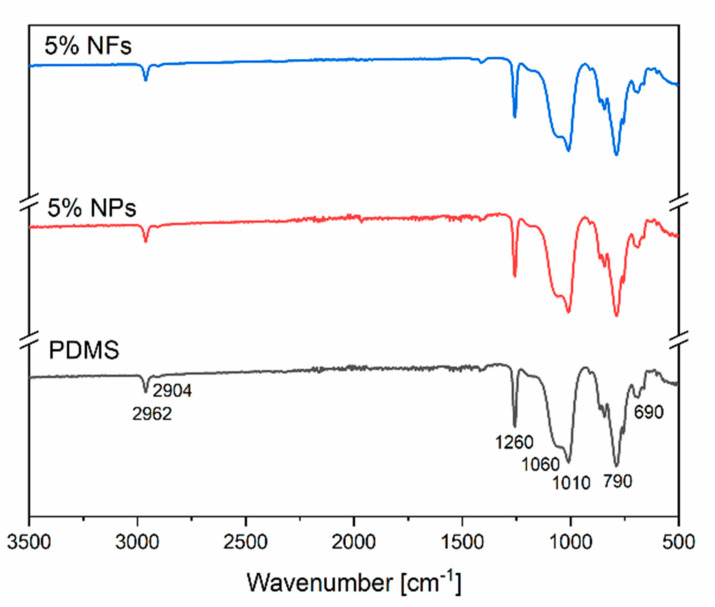
FTIR spectra of PDMS, PDMS-5% ZnO NPs and PDMS-5% ZnO NFs.

On the other hand, in order to study the interaction between the PDMS and the ZnO nanofillers, FTIR spectroscopy has been used. Figure 10 shows the FTIR spectra obtained from PDMS and PDMS with 5% wt fillers’ concentration, with the typical bands corresponding to a PDMS microstructure.

The vibrational bands at 2962 cm^−1^ and 2904 cm^−1^ refer to CH-stretching related to methyl groups CH_3_. The band observed at 1260 cm^−1^ indicates a CH_3_ symmetric deformation of Si-CH_3_ as well as CH_2_ wagging. Bands at 1060 cm^−1^ and 1010 cm^−1^ are related to Si-O-Si stretching vibrations commonly associated with siloxane structures. The bands at 790 cm^−1^ and 690 cm^−1^ are related to Si-C stretching and CH_3_ rocking vibrations. The 3D PDMS networks are formed by the curing process between the base polymer and the curing agent consisting of polymer chains with –O-Si-(CH_3_)_2_-O- repeating units together with methylene bridges. As mentioned in the previous section, the fabrication of PDMS-ZnO nanocomposites consisted of dispersing the ZnO fillers in the base polymer followed by adding the curing agent to the mixture, leading to the formation of a 3D PDMS network with embedded ZnO nanostructures. The hydroxyl groups on the ZnO nanostructures surface can react with SiH groups of the curing agent leading to the formation of extra Si-O-Si bonds. However, as shown in Figure 10, the spectra corresponding to unloaded PDMS and ZnO-PDMS nanocomposites (5% wt) are very similar (no detectable difference in peak intensity and the overall spectra) indicating that the chemical interaction between ZnO fillers and PDMS is limited.

### 3.3. Dynamic Mechanical Characterisation of PDMS-ZnO Nanocomposites

**Figure 11:**
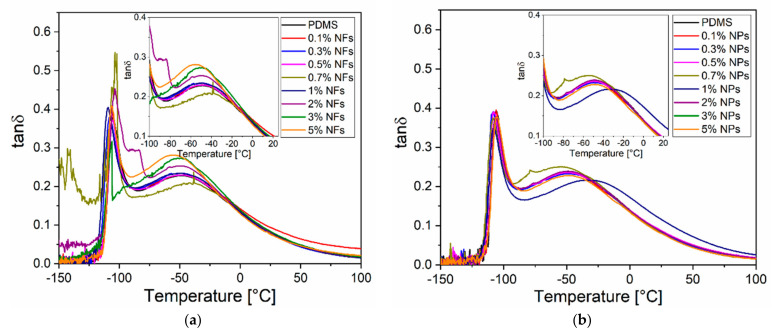
Tanδ as a function of temperature (inset: two peaks associated with T_g_ and Tm, respectively) of (**a**) PDMS-NFs and (**b**) PDMS-NPs.

DMA measures the response of material when applying an oscillating force or deformation to analyse the viscous and elastic contributions on the mechanical properties. These responses are expressed in terms of storage modulus (elastic modulus, *E*′), loss modulus (viscous modulus, *E*′′) and tan δ (damping coefficient). Storage modulus is generally related to the stiffness of the material and refers to the mechanical energy storage capabilities of the material while the loss modulus represents the dissipated heat (hysteresis) which is very sensitive to molecular motions, transitions, and relaxation processes. Tan δ is the ratio of the loss modulus to the storage modulus represented generally as a peak that can be related to the T_g_ of the material [45,46]. As the temperature increases, molecular chains started to move more freely and therefore, an increase in loss modulus was observed. As a result, the material became less stiff and more rubbery causing a decrease in the storage modulus. Therefore, the tan δ is associated to the glass transition temperature of the polymer (T_g_). The tanδ peak is usually related to the T_g_ which can give insight into the degree of interfacial interaction between the polymer matrix. Typically changes in T_g_ of the nanocomposites can be attributed to the type of interaction between the polymer and the nanofillers. In order to investigate the effect of the addition of the type and concentration of nanofillers on the thermomechanical properties of the PDMS-ZnO nanocomposites, a dynamic mechanical analysis under temperatures between −150 °C and 100 °C was carried out. Parameters such as tanδ and storage modulus were obtained. From Figure 11a,b, tanδ is observed to have a first predominant peak at about −107 °C and a second peak at about −50 °C for both unloaded PDMS and nanocomposites.

The first peak at approximately −107 °C is associated with the first transition of the segmental motion of PDMS chains corresponding to the glass transition temperature (T_g_). T_g_ has not been observed to shift dramatically by more than 10 °C as a function of filler concentrations for both cases which is indicative of poor polymer–filler interaction. The second peak at about −50 °C is attributed to the polymer chains located in the interphase region. However, this second peak is different as concentration increases in the case of NFs. The peak seems to become narrower for higher concentrations between 2 and 5% wt while at lower concentrations the peak seems broader. This behaviour is due to the fact that the volume fraction of the interphase region increases at higher concentrations, immobilizing the polymer chains around the fillers. On the other hand, the second peak corresponding to the NPs, did not show any trend as filler concentration increases [47,48].

**Figure 12:**
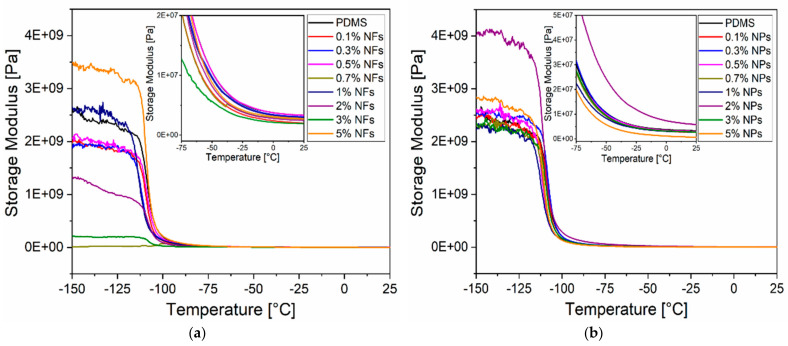
Plots showing: *E*′ (storage modulus) as a function of the temperature of (**a**) PDMS-NFs and (**b**) PDMS-NPs (inset: zoom of temperature ranges between −100 and 25°C).

Figure 12a,b shows *E*′ as a function of temperature for PDMS-NFs and PDMS-NPs respectively. *E*′ of nanocomposites remained in the same order of magnitude as neat PDMS regardless of the type of filler. However, *E*′ slightly decreased for NFs and NPs when compared to neat PDMS at higher temperatures including room temperature. These results indicate that the addition of ZnO NFs and NPs does not act as a reinforcement in the PDMS matrix at concentrations up to 5% wt, which once again, validates the flexibility of the nanocomposites.

### 3.4. Thermal Characterisation of PDMS-ZnO Nanocomposites

**Figure 13:**
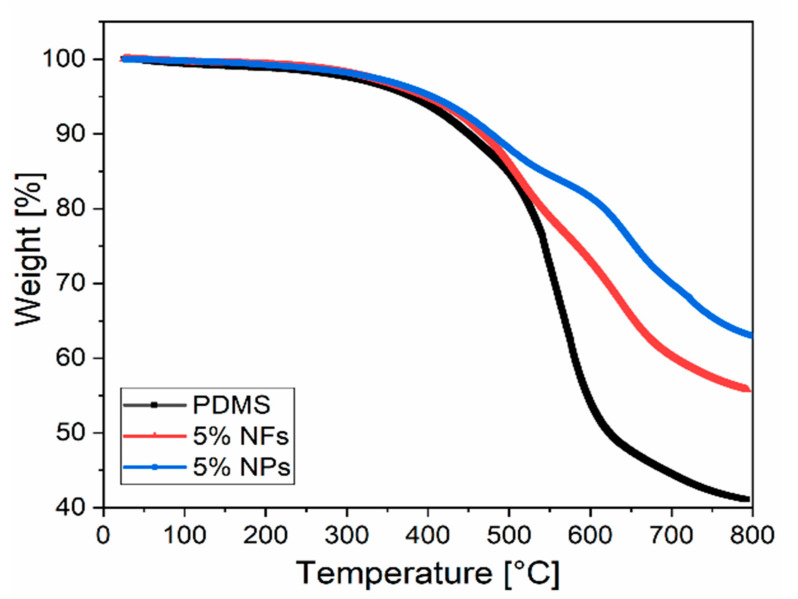
Thermal analysis curves for PDMS, PDMS-5%ZnO NPs and PDMS-5%ZnO NFs.

The influence of ZnO fillers on the thermal degradation of the nanocomposites has been investigated by thermogravimetric analysis. Figure 13 shows the thermal analysis curves upon heating of the PDMS and PDMS nanocomposites.

The decomposition temperatures at 5% and 20% weight loss and the residual weight at 800 °C for neat PDMS and PDMS nanocomposites at 1, 3 and 5 % wt concentrations are summarised in Table 2. A slight increase in thermal stability can be observed at 5% and 20% of weight loss when adding ZnO fillers into PDMS regardless of type and shape. On the other hand, the residual weight at 800 °C is approximately 56% for 5% wt NFs and 61% for NPs while for unload PDMS is around 41%. The difference in weight loss between the two fillers can be attributed to the size and geometry of the fillers, interphase region and presence of agglomerates. Therefore, the presence of larger agglomerates together within PDMS-NPs act as a barrier, delaying the degradation of the polymer chains compared to PDMS-NFs [49]. Overall, the incorporation of ZnO nanofillers provides higher thermal stability to the nanocomposite with respect to the neat PDMS, due to the high heat resistance and thermal stability of the ZnO fillers as reported in previous studies [20,21,49,50]. 

### 3.5. Piezoelectric Characterisation

**Figure 14:**
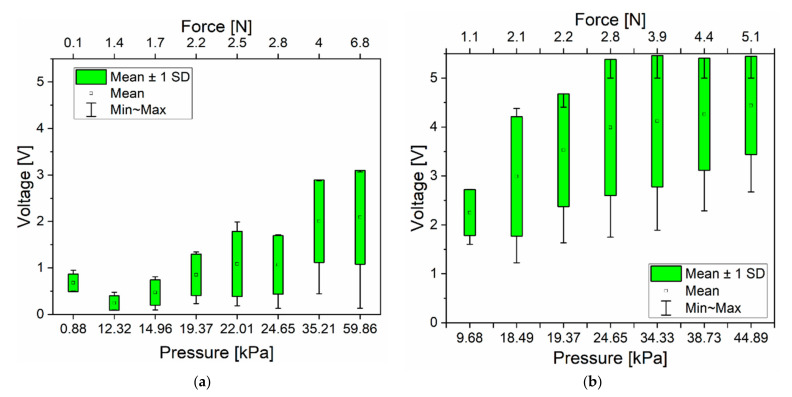
Piezoelectric response of the PDMS piezocomposites sensors (**a**) 10%-NPs and (**b**) 10%-NFs as a function of applied force.

The piezoelectric response of the nanocomposites was evaluated when subjected to a compressive force. It was found that a higher concentration than 5% wt ZnO was required in order to obtain a significant piezoelectric response. Considering that the mechanical response trend is favourable in this order of ZnO concentration, we present in Figure 14a,b the piezoelectric response of the piezocomposites at a concentration of 10% in weight for both geometries. The box-plot represents the standard deviation of the minimum and maximum measured voltage for each applied pressured ranging from 0.1 kPa up to 50 kPa.

The NFs piezocomposite generated 4.2 V while NPs generated 1.86 V under the same force of ca. 4 N (corresponding to a pressure of ca. 35 kPa). The data also show that the generated voltage increases with the increase in applied force regardless of the type of filler. In order to explain the effect of the filler geometry in the piezoelectric response, several factors need to be considered. Firstly, the crystalline 0002 planes of the ZnO nanostructures are responsible for the voltage generation when oriented perpendicular to the applied force. The flower-like structures exhibit more crystals in this direction due to the spatial distribution of their geometry compared to nanoparticles. In addition, several studies have reported that ZnO nanostructures such as nanoflowers and tetrapods are more susceptible to larger deformations (bending) under compressive force due to their high aspect ratio with respect to nanoparticles [10,24,32,51].

Another factor to consider is the surface area of the nanofillers. The NFs have a surface area of 2.2 m^2^/g while NPs have 13.994 m^2^/g (Table 1) which means that the decrease in particle size leads to more formation of agglomerates within the polymer matrix and the filler-filler interaction is stronger NPs. In this case, the electron flow might be trapped in the dipoles at the ZnO-ZnO interphase region and no connection path occurs to the electrodes, resulting in no piezoelectric response. Therefore, one could expect that the percolation threshold for NPs is higher compared to the NFs. Piezocomposites up to 5% wt did not show any piezoelectric response regardless of the type of filler. This result could be attributed to two reasons: firstly, concentrations under 5% wt might not produce enough volume occupied across the width of the material leading to no contact between the top and bottom electrode and secondly, not enough contribution from the volume of interphase regions. However, when the concentration is increased up to 10% wt, the volume occupied by the nanofillers increases, the formation of agglomerates increases and the contribution of the interactions of the interphase regions also increases causing a piezoelectric response under compressive load. Others have reported the need of using higher concentrations of ZnO nanofillers as well as employing alignment techniques (i.e., dielectrophoresis) or doping in order to obtain a piezoelectric response in a polymer matrix using different working modes [3,14,17,23,28,33].

On the other hand, sensitivity was determined by the linear relationship between the generated voltage and the applied force. The sensitivity of the piezocomposite was 59.3 mV/kPa and 21.6 mV/kPa for NFs and NPs nanocomposites, respectively. The difference in sensitivity might be due to the overall mechanical properties of the piezocomposites. Although the mechanical characterisation of the nanocomposites was performed up to 5% by weight, we have shown that the mechanical behaviour at this range of concentrations follows a consistent trend which is favourable to the targeting application; meaning that *E* is low and the *E* of PDMS-ZnO NPs is higher than that of PDMS-ZnO NFs at this concentration range. A higher stiffness results in a smaller piezoelectric sensitivity which can explain the different piezoelectric sensitivities observed for the 10% ZnO nanocomposites (NP vs. NF) in this work.

## 4. Conclusions

The fabrication and characterisation PDMS-ZnO nanocomposites show that the addition of the nanofillers did not constrain the flexibility and thermomechanical stability of the nanocomposites up to 5% wt which is favourable for the use of these materials in pressure sensors. However, it was found that there is a correlation between the filler concentration and the piezoelectric response from the PDMS-ZnO nanocomposites, hence, a higher concentration than 5% wt is required in order to obtain a piezoelectric response. The 10% wt NFs nanocomposite generated a response of 4.2 V while 10% wt NPs nanocomposite generated 1.86 V under an applied pressure of 3.5 kPa. The sensitivity of the nanocomposites was 59.3 mV/kPa and 21.6 mV/kPa for NFs and NPs, respectively, and the difference can be attributed to the fact that *E* for NFs is smaller than NPs, hence, the lower the stiffness the higher the sensitivity. In addition, NFs are more likely to be more sensitive to the applied pressure due to the spatial distribution and geometry of the fillers compared to the NPs. Therefore, in-house NFs can provide better performance, stability and robustness when used in nanocomposites for sensing applications compared to commercial NPs. The selection of the geometry of the nanofillers should be carefully selected in order to maximise the advantages when targeting specific applications, specifically if piezoelectricity is required. Overall, our results fully suggest the potential use of simple and low-cost PDMS-ZnO nanocomposites for pressure sensors exhibiting good mechanical flexibility, excellent thermal stability and biocompatibility by a simple and cost-effective fabrication process.

## Figures and Tables

**Table 1:** Summary of morphological characteristics of ZnO nanostructures.

Type	Shape	Particle Size [nm]	BET Surface Area [m^2^/g]	*d_BET_* [nm]
ZnO NPs	Rhombohedral	<100 nm	13.994	76.50
ZnO NFs	Flower	stick length ~3–6 μm diameter ~180–220 nm	2.200	486.58

**Table 2:** Summary of decomposition temperatures at different weight loss [%] for all samples.

	NFs			NPs		
Concentration [% *w*/*w*]	T5%	T80%	* T800%	T5%	T80%	* T800%
PDMS	379.75	526.84	41.06	379.75	526.84	41.06
1	418.89	527.06	22.26	404.09	543.70	52.95
3	403.55	544.17	48.98	408.13	574.07	57.69
5	398.01	542.38	55.76	405.11	617.25	60.63

* Residual weight at 800 °C.

## Data Availability

Not applicable.
